# Effect of a patient-centered drug review on polypharmacy in primary care patients: study protocol for a cluster-randomized controlled trial

**DOI:** 10.1186/s13063-015-0915-7

**Published:** 2015-08-26

**Authors:** Susann Hasler, Oliver Senn, Thomas Rosemann, Stefan Neuner-Jehle

**Affiliations:** Institute of Primary Care, University of Zurich, University Hospital Zurich, Zurich, Switzerland; Institute of Primary Care, UniversitätsSpital Zürich, Pestalozzistrasse 24, CH-8091 Zürich, Switzerland

**Keywords:** Multimorbidity, Polypharmacy, Shared-decision--making, Prioritization, Adverse drug events

## Abstract

**Background:**

Managing patients with polypharmacy is a challenging issue in primary care. The aim of this study is to determine whether a patient-centered systematic review leads to more appropriate medication use in patients without negatively affecting quality of life and the course of the disease.

**Methods/Design:**

The trial is a two-armed, double blinded cluster-randomized controlled trial. Primary care physicians (PCPs) will be randomly assigned to the intervention or control group. Physicians in the intervention group undergo training with instruction of the algorithm. The control group is given a lecture on multimorbidity and instructions for collecting data in a usual care manner.

PCPs will approach patients aged 60 years or older who are taking 5 or more drugs. The study period is 1 year.

The primary outcome measure is the change in the number of drugs 12 months after the algorithm was applied by the PCP during consultation with the patient. Secondary outcomes are: change in the number of drugs immediately after the encounter and 6 months later, reason for a change of the medication, discrepancy in the decision to change between PCP and patient, number of drugs for which the patient is suggesting a change, number of drugs the patient is taking that are not known to the PCP, time consumption of the intervention, disease-specific variables to evaluate the course of the disease(s) for which the patient is being treated , quality of life, barriers against using the algorithm, numbers of drugs readopted due to an unfavorable course of the disease, and numbers of drugs which have been started.

**Discussion:**

Answering the four questions of the algorithm requires a weighing-up of risks and benefits and contains a shared-decision-making approach: a prioritization of the treatment goals is necessary. This can only be done in collaboration with the patient. The majority of patients with multimorbidity are treated in the primary care setting. This underlines the significance of our study carried out in this setting: given the high prevalence of adverse drug events in patients with multimorbidity an intervention like ours has a large potential to reduce drug-related morbidity.

**Trial registration:**

ISRCTN16560559 13 November 2014

## Background

The intake of multiple drugs (polypharmacy), overuse and misuse of drugs are increasing problems in the care of people with a number of health conditions (multimorbid) as well as older people [1, 2]. One of the problems in polypharmacy is the danger of adverse drug reactions [3]. This leads to an increase in morbidity, hospital admissions [4, 5], health-related costs [6] and number of deaths [7, 8]. Paradoxically, a clear relationship between polypharmacy and underuse of indicated drugs has been shown [9–11]. Up to now, several clinical tools and approaches to guide appropriate prescribing of drugs are available [11-14]. Deprescribing is a complex process that is required for the safe and effective cessation of inappropriate medications [3]. Barriers to and enablers of deprescribing from the point of view of the patients as well as from primary care physicians (PCPs) have been investigated [3, 15–18]. A recently published study using a newly developed algorithm for a systematic reduction of medication (Good Palliative Geriatric Practice, GPGP) showed that deprescribing resulted in a significant positive effect on health in older patients [19, 20]. In our study, we slightly adapted the GPGP algorithm and pilot-tested the algorithm in a primary care setting to assess feasibility and practicability [21]. The algorithm enforces a systematic drug review considering patients’ perspective and preferences. As primary care physicians (PCPs) take care of the majority of patients with multimorbidity, our tool has a potentially high impact on polypharmacy in primary care settings. Previous studies, investigating interventions to reduce polypharmacy, have taken place in an older persons care setting. In primary care settings results are still lacking. This study intends to bridge this gap.

### Study hypothesis

The implementation of an algorithm adapted to the GPGP leads to a reduction of polypharmacy among patients with multimorbidity (60 years and older) in a primary care setting.

Furthermore, this implementation does not worsen the quality of life or the course of the disease for which the drug was originally prescribed for (safety issues of the intervention).

## Methods/Design

**Fig. 1:**
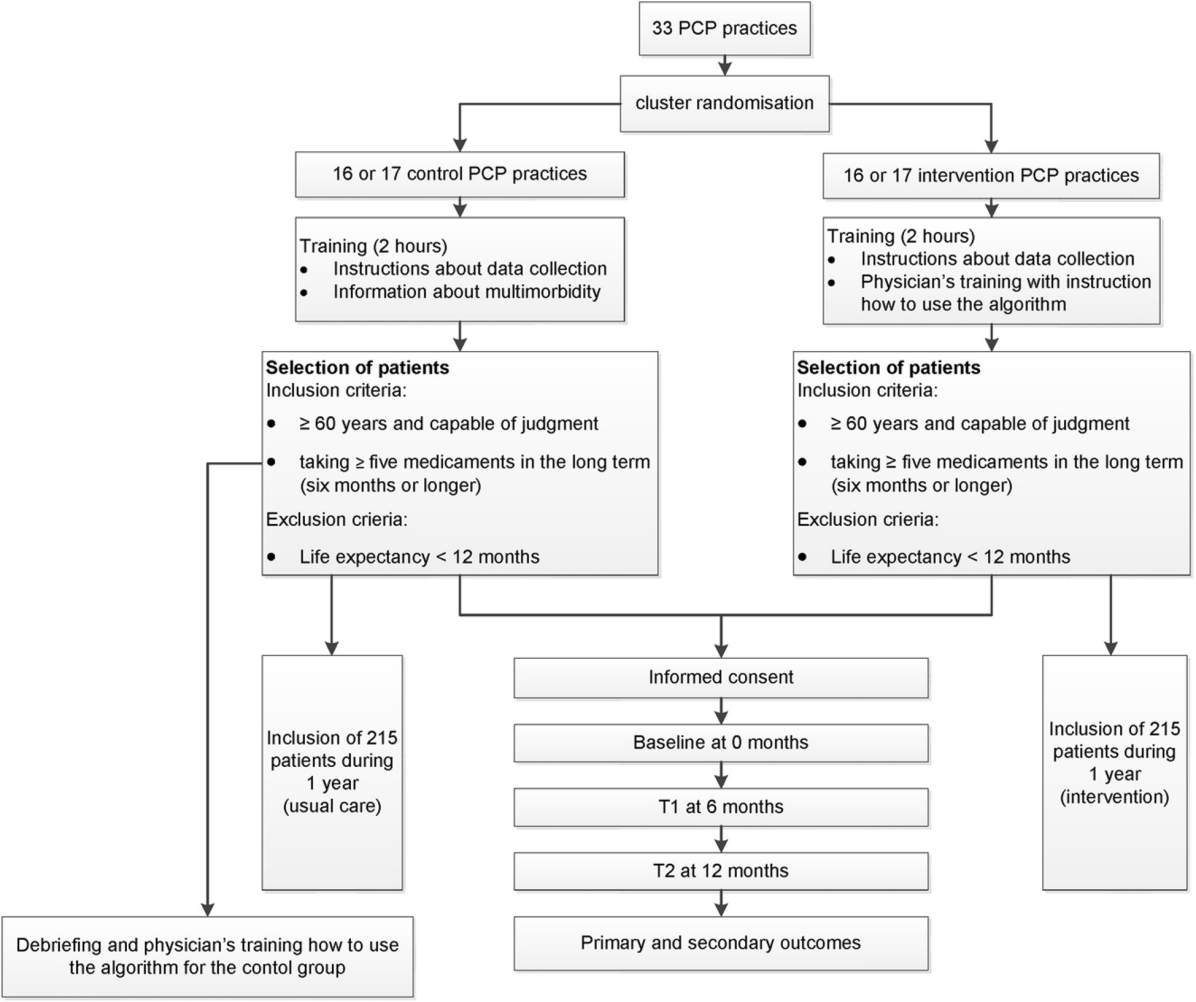
Study flow chart

We will conduct a cluster-randomized controlled trial (randomization on PCP-practice level) with primary care physicians in the northern part of Switzerland (see Fig. 1). The design and methodology of our study is based on the experiences of a small pilot study.

### Pilot study

The pilot study [21] was conducted with 14 primary care physicians. An evaluation was conducted to test the logistics, baseline measurements and the feasibility of the study. The pilot study resulted in minor changes in the questionnaire and an important element of shared-decision-making was added: beside the four major clinical problems defined by the PCP the four most important complaints in a patient’s perspective were added. In order to detect potential undertreatment a special section was added entitled “medication started”. 

### Recruitment and eligibility of primary care physicians

PCPs are eligible for participation if they provide care in the routine primary setting to unselected patients. About 1700 PCPs in the Canton of Zurich will be invited by a formal letter of the Institute of Primary Care of the University of Zurich.

PCPs who finally agree to participate will be listed in alphabetical order. If two PCPs of the same practice agree to participate, the first name in the alphabet receives a number which is valid also for the second one in order to avoid contamination between groups. Randomization will be stratified according to the practice size (e.g. single-handed versus group practice). The randomization scheme will be generated by using the website Randomization.com (http://www.randomization.com) and group assignment will be performed by a study nurse not involved in further data analysis. The PCPs with the corresponding numbers will be randomly allocated to the intervention and control group, respectively.

PCPs will be informed only after completion of the study about the group they have been randomized to (intervention or control group) in a debriefing session. At this time, an education session on how to use the algorithm will be offered to the control group.

If the number of the required 33 (see power calculation below) participating PCPs cannot be reached, the randomization will be done with the interested PCPs and the recruiting will be expanded to other Cantons of the northern area of Switzerland, followed by another randomization process.

Each PCP in the intervention and control groups will receive a financial allowance.

### Patient recruitment

All PCPs are asked to approach, consecutively and regardless of the reason for the current consultation, patients aged 60 or older who are taking 5 or more long-term drugs.

### Patient inclusion criteria

Be aged at least 60 years.

Be taking 5 or more long-term drugs (6 months or longer).

### Patient exclusion criteria

Life expectancy less than 12 months.

### Intervention

Blinding of PCPs is not possible in this study design and even participation in a study addressing a certain topic can influence a physician’s behavior. To achieve a degree of blinding, PCPs are not provided with specific information at invitation. They will receive a lecture (length: 2 hours) about multimorbidity in general and instructions for collecting data in a usual care group. PCPs from the control group will be told that the study purpose is to investigate best practices for physician-patient communication.

**Fig. 2:**
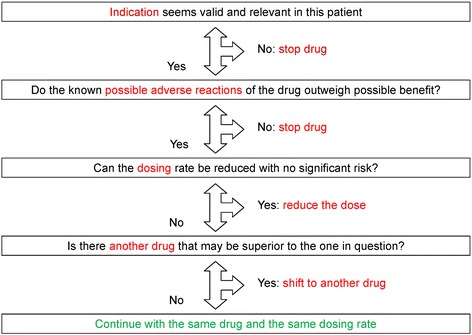
Improving drug therapy in primary care (adapted from [20] and [21])

PCPs in the intervention group will undergo a physicians’ training with instruction on how to use the algorithm including a communication skills training. The adapted GPGP-algorithm used in our trial consists of four steps and is a patient-centered process. First, the drugs are listed together with the four main diagnoses as well as the four main disorders from the patient’s perspective. Then the following steps will be systematically followed (see Fig. 2):

1. Indication of the drug in this patient (identifying undertreatment or overtreatment)
2. Do the known possible adverse reactions of the drug outweigh the possible benefits? (Potential adverse events)
3. Can the dosing rate be reduced with no significant risk? (Dosing problems)
4. Is there another drug that may be superior to the one in question?

Finally, the result of this analysis will be discussed together with the patient. For each drug the PCP will note whether the patient agrees with the recommendation.

After obtaining informed consent from the patient, a practice nurse or the PCP creates a list of the patient’s present medication. Then, the PCP defines the four major clinical problems and, together with the patient, the four most important complaints from the patients’ perspective. Physicians in the intervention group then decide, for every drug listed, whether the indication is correct, whether there are side effects, whether an alternative treatment would be suitable or whether a change in the dosage is indicated (key questions of the algorithm). After discussion with the patient, the PCP and patient decide together whether to stop a drug, to change the dosage of a drug or to switch to an alternative drug, with the option to restart if symptoms should increase or the disease deteriorate.

### Primary outcomes measures

Change in the number of drugs (deprescribing rate) 12 months after applying the tool.

The primary outcome will be calculated on the basis of the medication list, provided by the practice nurse or the PCP at baseline (before the intervention) and 12 months afterwards.

### Secondary outcome measures

Table 1 displays the secondary outcomes with the appropriate measuring method.

**Table 1:** Secondary outcomes and the appropriate measuring method

Secondary outcomes	Measuring method
1. Change in the number of drugs immediately after the encounter and 6 months later	Medication list at baseline and after 6 months
2. Reason for a change, categorized in the 4 options of the algorithm, number of drugs in each category 12 months after applying the tool	Medication list of the changed medications after the intervention, with the reason for a change
3. Discrepancy in the decision to quit, change or continue the drug between PCP and patient	Medication list after intervention with any suggestion from the PCP for a change; medication list after the intervention and after shared-decision-making
4. Number of drugs for which the patient is suggesting a change	Medication lists at baseline, after 6 and 12 months
5. Number of drugs the patient is taking not known to the PCP	Medication lists at baseline, after 6 and 12 months
6. Time consumption of the intervention	Measurement of the time through the practice nurse and the PCP
7. Disease-specific variables to evaluate the course of the disease(s) for which the patient is being treated	Symptom scores and measurements of biometric analysis (e.g. blood pressure monitoring, serum glucose) and VAS scales (e.g. pain). Event rates (hospitalization, death) and unexpected adverse event rates
8. Number of drugs readopted due to an unfavorable course of the disease(s) (readoption rate)	Medication lists at baseline, after 6 and 12 months
9. Quality of life (QoL)	Patient rating on a 5-point Likert scale
	EQ-5D-3 L at baseline, after 6 and 12 months
10. Barriers perceived by patients and PCPs against the approach/algorithm	Phone interview with the patients 13 months after intervention
11. Number of drugs which have been started	Medication lists at baseline, after 6 and 12 months

*EQ-5D-3 L* EuroQol health status measure, *PCP* primary care practitioner, *VAS* visual analogue scale

After the baseline assessment (including gender and living situation) systematic follow-up measurements will take place after 6 and 12 months.

#### Patient-reported outcomes

Quality of life (QoL) will be assessed using the EuroQol (EQ-5D-3 L). This is a generic preference-based health status measure that has been shown to be valid and reliable in a variety of populations and patients groups [22–25].

Furthermore, patients are asked to determine their QoL on a Likert scale from −2 to +2. They are asked about their main complaint and they have to indicate the intensity (no complaint to unsupportable) on a visual analogue pain scale.

#### Disease-specific parameters

PCPs of both groups measure the following disease-specific parameters at baseline, after 6 and after 12 months:

- Weight and blood pressure
- Glycated hemoglobin (HbA1c), for patients with diabetes only
- Thyroid-stimulating hormone (TSH), for patients with thyroid hormone substitution only
- Hemoglobin, for patients with iron substitution or vitamin B_12_ substitution only

Furthermore, the PCP evaluates the course of the main diseases (stable, improvement, aggravation).

#### Safety outcomes

Adverse events have to be reported by the PCP at the study center and the safety board. The following definition for adverse events is used:

- Complication of an existing disease (e.g. myocardial infarction in a coronary heart disease patient)
- Acute illness newly diagnosed
- Hospitalization or death

In case of adverse events the safety board can consult the medical history of the patient and, together with the PCP, it decides whether there is a plausible connection between intervention and adverse event.

The safety board is composed of a clinical ethicist, biostatistician and a geriatrician.

### Data collection procedures

Patients will receive detailed written information on the aim of the study. After giving their written informed consent data will be collected by means of paper documents. For every patient a dossier with the different case report forms will be created. The forms are encoded; the code is stored at each primary care physicians’ practice. If a case-tracking is needed (in case of adverse events) it can be decoded. The transfer from paper to electronic data will be carried out through a research associate and controlled by a second research associate. Grouping of diagnoses and drugs will be organized by standardised databases.

### Statistical analysis

The primary data analysis will follow the intent-to-treat (ITT) approach. This means that all available data from all individuals will be analyzed according to treatment group assignment, regardless of whether or not each individual actually received the assigned treatment.

The primary outcome, change in the number of drugs 12 months after applying the deprescribing tool, will be compared by using a *t* test for independent groups comparison. Hierarchical regression will be used to consider the cluster-design for potential confounder. Determinants associated with a change of the medication are investigated by exploratory, multivariate regression analysis.

For secondary outcomes, parametric (*t* test) or non-parametric tests (chi-square and Wilcoxon test) will be used where appropriate.

Descriptive statistics will be used to describe the study population. Drop-out and loss to follow-up will be described.

In case of study discontinuation the collected data that are hitherto available will be anonymized and evaluated.

The last observation carried forward (LOCF) method will be used for dealing with the missing data for patients who have dropped out. The drop-out rate and the causes of drop-outs will be compared between the two groups to investigate the possible influence of the intervention. To estimate the last observation carried forward-effect a per-protocol (PP) analysis will be applied.

### Timeframe of the study

The recruitment of the PCP is planned over 2 months between March and May 2015.

Patients’ eligibility screening and patient inclusion is projected within a period of 4 months. The aim is to include 1 patient/week per PCP.

### Ethics approval

The study protocol is approved by the Ethics Committee of the Canton of Zurich (reference KEK-ZH-number 2015–0595).

### Patient informed consent

Previous to study participation, patients receive written and verbal information about the content and extent of the planned study from the PCPs. In case of acceptance, they sign the informed consent form.

### Data security/disclosure of original documents

The patient names and all other confidential information fall under medical confidentiality rules and are treated according to appropriate Federal Data Security Laws. The patient names are not accessible to the study staff.

### Sample size calculation

Assuming a 0.05 2-sided significance level, 30 clusters (on PCP-level) including 13 patients each would have an 80 % power to detect a difference of 1 drug, which we consider to be clinically relevant.

Based on previous studies [14] we assumed an intracluster correlation coefficient (ICC) of 0.02 for the primary outcome and a standard deviation of 2.8 [21]. In consideration of a drop-out rate of 10 %, 33 PCPs (16 respectively 17 PCPs with 215 patients in each arm) are needed.

## Discussion

The aim of this study is to determine whether a systematic review with a specific algorithm leads to more appropriate medication use in patients with multimorbidity (60 years and older) without worsening their quality of life and the course of the disease. Up to now, similar interventions have been tested in older populations but not in a primary care setting. In this setting, it is crucial, that an intervention is time-saving for enabling implementation in the daily clinical practice. In the pilot study the expenditure of time was 15 minutes (median; interquartile range (IQR): 10–30). The feasibility and acceptability was rated with a 5-point Likert scale in different items (means: 3.2–4.2, SD 0.83–1.3). Other studies concerning improving appropriate prescription in (mostly older) patients receiving complex polypharmacy present a more time-consuming algorithm or method than usual care [26]. The time aspect might be a strength of our approach in comparison to other ongoing studies [14, 15, 24].

A special feature of our study is the investigation of overtreatment as well as undertreatment. After piloting the study, an extension was made: beside the list of medications in use a special section was added with “medication started.”

Prioritization of patient’s problems is a crucial issue in the shared-decision-making process. To foster this approach and discussions between physician and patient on this issue, we added a list of the four most important complaints from the patient’s perspective to our tool in in parallel with the list of the four major clinical problems defined by the PCP.

Based on other studies [24], measurements of EQ-5D-3 L might even enable an economic evaluation of the danger of adverse drug reactions.

Finally, our study contains an exploratory part, as patient’s barriers and enablers against the approach will be inquired systematically 13 months after intervention. This has the potential to optimize the algorithm or further adapt it to the specific needs of primary care.

### Trial status

Patient recruitment had not started at the first submission in April 2015.

## Abbreviations

GPGP: Good Palliative Geriatric Practice
HbA1c: glycated hemoglobin
ICC: intracluster correlation coefficient
IQR: interquartile range
ITT: intent-to-treat
LOCF: last observation carried forward
PCP: primary care physician
PP: per-protocol
QoL: quality of life
SD: standard deviation
TSH: thyroid-stimulating hormone
